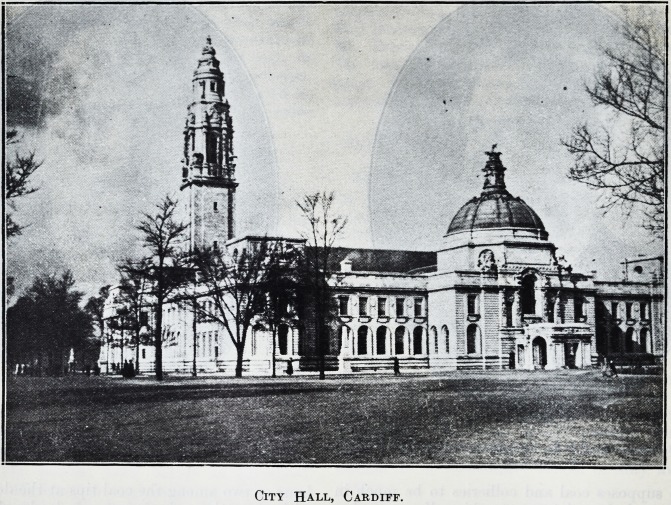# Public Health: Interviews with Local Authorities: Cardiff

**Published:** 1924-05

**Authors:** 


### THE PUBLIC HEALTH.

### INTERVIEWS WITH LOCAL AUTHORITIES.

### XVII.—THE CITY OF CARDIFF.

On his first visit to the Welsh capital the visitor is apt to find that he has fallen into error. We are not thinking of the sort of crude mistake made by the gentleman who, having obtained his first impression of the Welsh tongue in a dockside restaurant, found that he had been listening to the conversation of Norwegian sailors. Rather are we referring to some of those preconceived notions of which the visitor has to dispossess himself as soon as he begins to look around and make enquiry. Thus, he supposes coal and collieries to be much in evidence, and he pictures to himself a murky, smoke-begrimed town, a sort of overgrown colliery village. And lo! a clean well-nigh smokeless city, with the nearest coal pits nine or ten miles away in the valleys beyond the low-lying range of hills to the north of Cardiff. Again, he presupposes slums in abundance as in other towns which have attained commercial prosperity during the nineteenth century. He learns that slums are non-existent. If on a mission of enquiry such as ours he anticipates that Welshmen only are to be found at the centres of authority in this land of intense passionate nationalism, he will again be wrong.

Dr. James Robinson, C.B.E., Chairman of Cardiff Health Committee.

[*Lafayette, Glasgow.*

Dr. R. M. F. Picken, Medical Officer of Cardiff.

### The Chairman and the Medical Officer.

For example, Alderman James Robinson, C.B.E., the Chairman of the Health and Port Sanitary Committee, does not bear a peculiarly Welsh name. But the people of Cardiff find in this gentleman, who is a general practitioner, and a very busy one, too, so very satisfactory a representative that they have placed him on their Health Committee for 23 years, during 17 of which he has adorned the chair. For their Medical Officer of Health Cardiff has gone north of the Tweed, and is to be congratulated on having so efficient a public servant as Dr. Ralph M. F. Picken.

### No Smoke.

We have said that Cardiff is a clean city. Smoke abatement is not one of the troubles of the Health authority. Millions of tons of coal come into Cardiff in the course of a year, but they pass it on. An hour or two among the coal tips at the docks, where, with a rattle and roar, truck loads of coal were emptying their contents with extraordinary ease into the holds of great ships, gave us some small indication of the quantity of coal that passes through Cardiff.

### The City's Extraordinary Growth.

Cardiff, of course, owes its prosperity to coal. The fine block of public buildings on the magnificent site adjoining the Castle—surely one of the finest sites of its kind in Europe—stands all gleaming white, paradoxically a monument to the black product which has made Cardiff; not the smoke-begrimed building which symbolises municipal progress in so many industrial areas. The beginnings of the Welsh coal industry are to be traced back less than a hundred years. If we go back a little further to the year 1811 we find Cardiff with a mere handful of people—2,577 to be precise. Then follows an extraordinary growth—in the year 1841, a population of 10,077; in 1881, 85,371; 1901, 164,333; and 1921, 203,700, to which has to be added some 20,000 for an area taken into the city by boundary extensions in 1922, which practically doubled its area and afforded new land much needed for the erection of houses according to modern ideas of town planning.

### The Housing Problem.

The statement that slums are non-existent must be accepted in the sense indicated by Dr. Johnson when he said that a remark that there are no apples in an orchard is not to be refuted because a diligent search might reveal an apple here and there. Though there are no slum areas, the Health authority nevertheless recognise that there is great over-crowding. The population of Cardiff has increased since 1911 at a more rapid rate than any other town with a population over 200,000. On the standard densities accepted for county boroughs the number of occupied rooms in Cardiff was 16 per cent. in excess of minimum requirements in 1911. There has been a falling away from this position to 1 per cent. below standard; stated in other terms, the excess of families over houses has increased by well over 4,000. The housing question in Cardiff, in one serious aspect, is not of recent creation. The tendency in the past, owing to heavy ground rents, has been to erect relatively large houses, and it is a fact and a matter of anxious consideration to the Health authority that nearly half the families in Cardiff are living under the conditions of multiple tenancy. The abolition of this evil must, it is recognised, be a gradual process. The remedy is twofold. Not only must there be building of new small houses, but it is thought that many of the relatively large number of houses of eight and nine rooms might be remodelled and converted into structurally separate houses. The deficiency already existing and to be overtaken within the next 10 years may be put at 10,000 houses. A good beginning has been made with the Council scheme on land taken into the City boundaries, but the Health authority is not committed to any theoretical view as to the best means of obtaining houses so long as they are produced by one means or another. One thousand houses a year for ten years should not be beyond the capacity of this great community, and there is a chance for Cardiff to give a proud lead to her English neighbours in the matter.

### A Well Co-ordinated Administration.

The city is well served by its Health Officers, whose work is co-ordinated within the limits at present possible. Not only the general health and sanitary work of the city, but also the important Port sanitary work are under the guiding hand of Dr. Picken. He is also School Medical Officer. It is a feature of his administration that the duties of separate services, closely interrelated, are combined under one Assistant Medical Officer. Thus, Dr. Herbert Sheasby is responsible for part of the School Medical Service, Port administration, venereal disease work and the medical inspection of aliens; Dr. Mary Adams for Maternity and Child Welfare and Mental Deficiency work, while Dr. Lilian Griffiths Dr. Harold Coultpard and Dr. Helena Webster combine School Medical and Maternity and Child Welfare work. The Chairman of the Health Committee is quite in accord with the view that to secure the greatest efficiency all the Health services in the city, including those for which the Guardians and the National Health Insurance authority are responsible, must ultimately be brought by legislation under one effective control. The Medical Officer paid tribute to the efficiency

City Hall, Cardiff.

of the sanitary inspecting staff. The public, he said, have become so accustomed to the work upon which this staff is engaged, which is the oldest branch of public health administration, that there is danger of its value failing to be recognised. It comprises the sanitary inspection of dwelling-houses and factories, getting the day-to-day defects remedied, the inspection of shops under the Shops Acts, the inspection of seamen's and common lodging-houses. It is to these officers, too, that the public have to look for protection from rotten and adulterated food. The system of food inspection in Cardiff is greatly facilitated by the absence of private slaughter-houses in the city, all animals being slaughtered in the public abattoirs belonging to the Corporation.

### Milk and the Small Shopkeeper.

Routine sampling of milk for bacteriological examination has been carried out for many years. The continued postponement of many of the valuable provisions of the Milk and Dairies (Consolidation) Act is a matter viewed by the Medical Officer with regret. The public should appreciate the need for increased stringency ; only 11 per cent. of the milks sampled during the hot months of the year were up to the standard laid down for grade A milk. Dr. Picken pointed out that there are over 400 vendors of milk in Cardiff, a great many of whom are small shopkeepers who sell milk, not as their staple trade, but to bring customers into their shops. These are, almost without exception, unsuitable for the sale of milk.

### The Baby under Four Weeks.

The marked decline in infant mortality to be noted in Cardiff, as elsewhere, in the last ten years, is naturally a source of great satisfaction to those immediately concerned. In regard to this, however, the Medical Officer wisely, if somewhat unusually, utters a note of warning. It is not safe to adopt too sanguine a view on the basis of an experience of years in which there has been an absence of those epidemic forms of disease which take especial toll of children ; it is not by any means certain that infantile diarrhœa is not an epidemic disease, subject to waves of increased virulence over periods of years. The baby under four weeks remains in any case a serious problem, no less than 36 per cent. of the deaths under one year belonging to this period of life. The medical staff need all the support and sympathetic interest possible from the Health Committee and the public in the work of the ante-natal clinics and in the educational work among mothers and expectant mothers carried out under the able supervision of Dr. Adams.

### The Growing Child.

The work in connection with Child Welfare reveals the serious need for hospital accommodation, both for children suffering from acute diseases of childhood and also for those who are convalescent in unsatisfactory surroundings. The Chairman of the Health Committee referred to the provision of a new small-pox hospital in delightful surroundings for which plans are well advanced, and expressed the hope that small-pox would be so good as to leave the people of Cardiff alone and make the proposed accommodation available for the youngsters. It is the merest truism that the whole welfare of the town of the future is dependent on the care of the child of to-day. With regard to the enormously important School Medical service, no more significant statement of the need for it could well be forthcoming than that which points out that of 4,000 children beginning school life in 1922, 32 per cent. were found to be so diseased or defective as to require medical treatment or observation.

### Port Work.

With some 3,000 foreign and 5,000 coastwise vessels coming into the port during the year, there is abundant work to be performed by the Medical Officers and the Sanitary Inspector, often at short notice and at very inconvenient hours. Constant vigilance is necessary as to the general sanitary condition of vessels, and close observation of seamen suffering from illness which may prove to be an obscure form of one of the dangerous infectious diseases. War has to be raged on the ship's rat—in passing, we note that there is a distinct sub-species of the *rattus rattus* which is known as the *rattus rattus rattus* which it is to be hoped does not harbour the plague flea in the generous proportion which the name would seem to indicate. The useful work performed by the inspecting officers has resulted in a great improvement in the sanitary condition of the floating population. Attached to the Port Clinic are ten beds always available in a modern hospital for seamen, and away on Flat Home Island in the centre of the Bristol Channel is accommodation in readiness, but fortunately rarely required, for cases of cholera, yellow fever or plague.

### The Importance of Education.

We cannot do better than conclude this short review of the work of the Cardiff Health Authority than by laying stress upon the importance which Alderman Robinson and his Committee, as advised by Dr. Picken, attach to education and propaganda. The limit to public health progress along established lines of administration, says Dr. Picken, seems almost to have been reached and further advancement will depend more largely on the enlightenment and co-operation of the individual members of the community than on new measures or improvements in the machinery. To which we may add that the citizens of Cardiff, for their part, may feel assured that the machinery will be kept in good running order by their Medical Officer and his staff.

---

### The Scialytic Light.

The Barbier, Benard and Turenne Scialytic Light is a high power automatic light, which can be especially recommended for use in operating theatres, laboratories, and dentists' rooms. This useful fitting, which consists of a dioptric lens in the centre of a metal saucer round the rim of which are a number of silvered reflectors, throwing an inverted cone of concentrated light on to the table. The fitting, which can be used in connection with electric or gas lamps, is remarkable in that it gives an entirely shadowless light and eliminates heat, glare and all head shadows. It can be fitted either as a fixed light or as one which may be tipped to any angle, and it will be found a great advantage to dwellers in districts where there is no electric light that special battery sets can be supplied with it. Any further information can be obtained from Major J. P. Ashley Waller, Audrey House, Ely Place, E.C. 1.

## Figures and Tables

**Figure f1:**
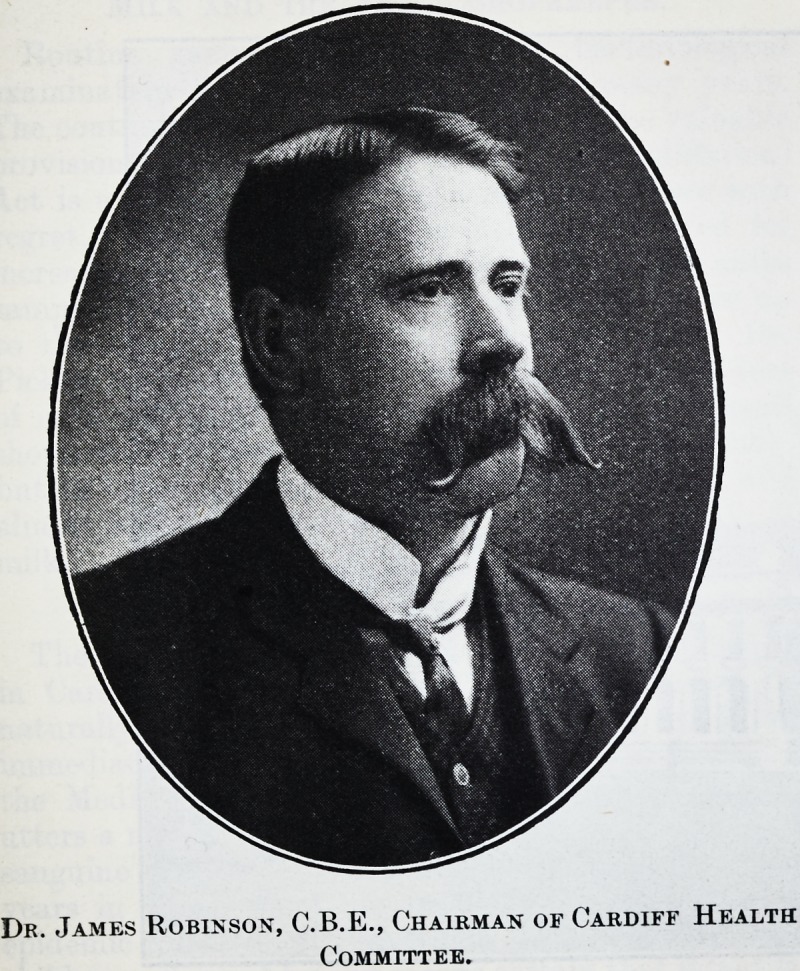


**Figure f2:**
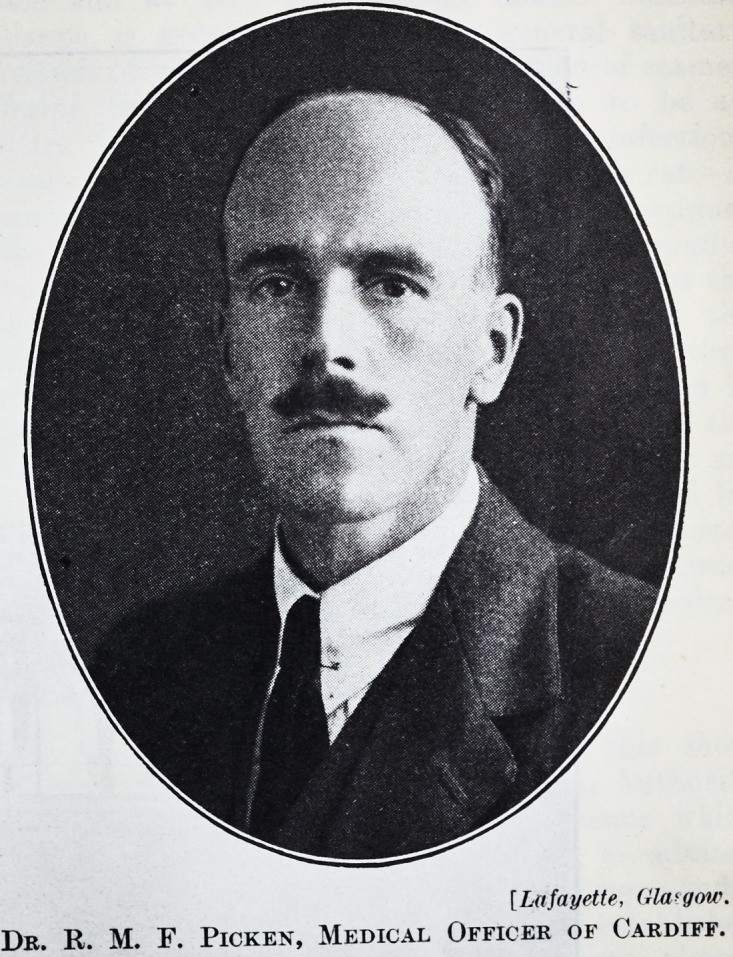


**Figure f3:**